# A Colony of Seven Hundred Lunatics

**Published:** 1843-06

**Authors:** 


*A Colony of Seven Hundred Lunatics.*—At the late sitting of the French Academy of Sciences a paper on Insanity was read by its author, Moreau, one of the physicians present. The chief object of this pamphlet is to recommend the adoption in France, as regards pauper lunatics, of the plan resorted to in Belgium.

Moreau states that in the village of Gheel, in that country, there is a colony of not less than seven hundred lunatics, who are treated upon so admirable a system that they are perfectly harmless, and live and labor with the same inhabitants, whose habits they acquire, and to which they become quite so attached that when cured they are frequently unwilling to quit the place. These lunatics are made useful in agriculture and manufactures, and consequently their cost is small as compared with that of ordinary lunatic asylums. The origin of this colony dates so far back as the sixth century, and is another verification of the old adage that there is nothing new under the sun! The mode of treating the lunatics at Hanwell, near London, was considered, when first put in practice, as a novelty, and yet it is nothing but Gheel practice imperfectly carried out. It is only surprising that this improved mode of treatment should have been deferred so long in England; and it is now evident that it is capable of great extension in its application. Within the last few years only, in that country, medical men have ascertained the possibility of so classing and occupying lunatics as to render even the most violent of them comparatively tranquil, and thus facilitating the curative process. For chains, whips, and other means of coercion, kindness and intelligence on the part of the keepers have been substituted, not only at Hanwell, but also at the Bethlehem Asylum.

